# Twists and turns in the story of learned avoidance

**DOI:** 10.7554/eLife.109427

**Published:** 2025-11-11

**Authors:** Lesley T MacNeil

**Affiliations:** 1 Department of Biochemistry and Biomedical Sciences, the Farncombe Family Digestive Health Research Institute, and the Michael G DeGroote Institute for Infectious Disease Research, McMaster University Hamilton Canada

**Keywords:** *Pseudomonas aeruginosa*, transgenerational epigenetic inheritance, learned pathogen avoidance, reproducibility, *C. elegans*

## Abstract

Evidence that learned avoidance of a pathogenic bacterium can be transmitted to future generations in *C. elegans* is growing.

**Related research article** Akinosho A, Alexander J, Floyd K, Vidal-Gadea AG. 2025. Independent validation of transgenerational inheritance of learned pathogen avoidance in* Caenorhabditis elegans*. eLife **14**:RP107034. doi: 10.7554/eLife.107034.

The ability to recognize and avoid pathogens is essential for survival. In some cases, animals recognize molecules produced by pathogens, allowing them to mount an immediate response. In other cases, animals learn to avoid the pathogen after they have been exposed to it – a phenomenon that is called “learned avoidance”.

The worm *Caenorhabditis elegans* can learn to avoid *Pseudomonas aeruginosa*, a pathogenic bacterium that causes disease in a range of species (Zhang et al., 2005). In 2019, Coleen Murphy and colleagues at Princeton University observed that learned avoidance of the PA14 strain of *P. aeruginosa* could be transmitted for up to four generations of *C. elegans* without new exposure to PA14 (Moore et al., 2019; Kaletsky et al., 2020). This transgenerational epigenetic inheritance allows animals that have never encountered PA14 to benefit from the experiences of previous generations.

Recently, Craig Hunter and colleagues at Harvard University questioned the inheritance of learned avoidance to the F2 generation (Gainey et al., 2025). While they observed learned avoidance to PA14 in parents (P0) and their progeny (F1), they did not observe avoidance in the F2 generation. The Murphy group responded, contending that the inability to observe transgenerational epigenetic inheritance was due to changes the Hunter group made to the original experimental protocols (Kaletsky et al., 2025). Now, in eLife, Andres Vidal-Gadea and colleagues at Illinois State University – Aalimah Akinosho, Joseph Alexander and Kyle Floyd – report that they have confirmed findings from the Murphy group by showing that learned avoidance of PA14 is passed on to the F2 generation (Akinosho et al., 2025).

In a standard avoidance assay, test spots of bacteria are placed at opposite ends of an agar plate, and sodium azide – a chemical that immobilizes worms – is added to each test spot (Figure 1). The worms are placed at the center of the plate and allowed to roam, and the number of worms in proximity to each test spot is scored after one hour (Moore et al., 2019).

**Figure 1.**
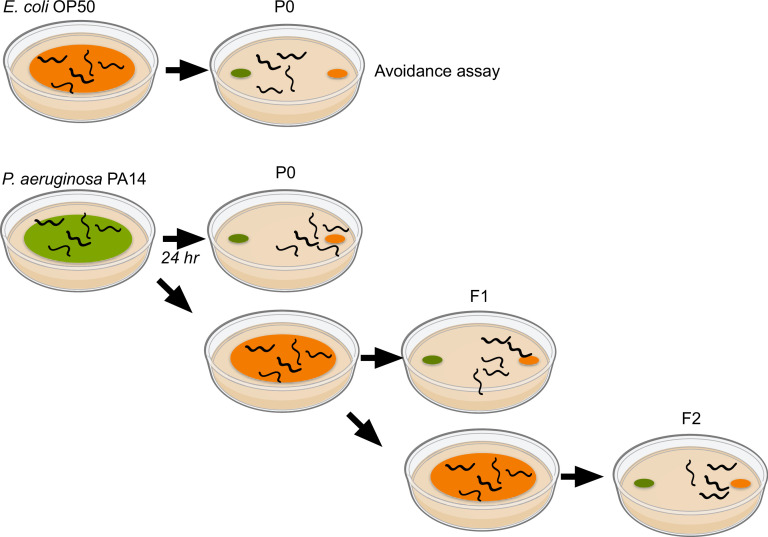
Transgenerational inheritance of learned avoidance of pathogens. An avoidance assay (top row) measures the ability of *C. elegans* worms to avoid specific bacteria. The worms are first grown on OP50 (orange; top left), a non-pathogenic strain of the bacterium *E. coli* that is the standard laboratory diet of *C. elegans,* before transfering them to a test plate. Spots of each bacterium are placed at opposite ends of a petri dish (top right), the worms are allowed to roam, and their position after a set period of time is recorded. When worms grown on OP50 encounter the pathogenic PA14 strain of *P. aeruginosa* (green) for the first time, they are initially attracted to it (top right). However, when worms are exposed to PA14 before the avoidance assay, they avoid PA14 during the assay (second row). This learned avoidance can be passed on to the next generation (F1; third row) and the generation after that (F2; fourth row), without these worms needing to encounter PA14.

For the learned avoidance assay, the worms are exposed to PA14 for 24 hours, a process sometimes called “training”, after which time they are collected and washed. A subset of these worms is transferred to a test plate and allowed to choose between PA14 and the OP50 strain of *E. coli* (which is the standard laboratory diet of *C. elegans*). This is the parental generation, and the worms in it will avoid PA14.

Eggs (F1) are collected from the remaining worms and transferred to standard OP50 plates. Once they reach the adult stage, the same process is carried out; a subset of animals is tested, and eggs are collected from the remaining worms for the next generation (F2). Importantly, after the parental generation, worms do not encounter PA14 before testing, but they retain the learned avoidance of the parental generation. To measure learned avoidance, all groups are compared to animals whose predecessors have never encountered PA14.

Both the Murphy and Vidal-Gadea groups used sodium azide in their assays to immobilize the worms, while the Hunter group immobilized them by lowering the temperature to 4 °C at the end of the assay. With azide, worms that reach the test spots before the end of the assay are immobilized. By contrast, when the temperature shift method is used, worms can come into contact with the test spots and then move away before the end of the assay. The Murphy group proposed that the use of azide ensured that animals were captured in their initial response, which prevented them from learning to avoid PA14 after encountering it during the assay. However, they argued, when the temperature-shift method is used, worms can encounter PA14, learn from this encounter, and avoid the PA14 spot (Kaletsky et al., 2025). Put simply, an encounter with PA14 could unintentionally introduce another source of learned avoidance to the assay. The most significant impact of this would be on the negative control, where it is assumed that the worms’ response reflects that of animals that have never encountered PA14.

Worms that have not previously encountered PA14 are initially attracted to it (Zhang et al., 2005). While the Murphy group consistently observed this attraction in their assays, the Hunter group generally did not (Kaletsky et al., 2025). The Vidal-Gadea group also observed that worms that had not been exposed to PA14 were initially attracted to it, suggesting that this is an important piece of the puzzle (Akinosho et al., 2025). Indeed, when tested directly, the Murphy group did not observe attraction using the temperature-shift method (Kaletsky et al., 2025). However, whether the omission of azide alone explains the discrepancy between the studies is not clear. In a handful of assays, the Hunter group used azide but failed to see the initial attraction to PA14, or to observe learned avoidance in the F2 generation.

P11, a small RNA produced by PA14, is necessary and sufficient to induce transgenerational epigenetic inheritance (Kaletsky et al., 2020). In addition, loss of P11 reduces chemoattraction to PA14 by reducing ammonia production (Marogi et al., 2024). The Murphy lab proposed that suboptimal P11 expression could explain difficulties in reproducing transgenerational epigenetic inheritance and in observing the initial attraction to PA14. Although they did not measure P11 levels, the Hunter group argued that their ability to observe learned avoidance in the F1 generation excluded the possibility that P11 expression was insufficient. PA14 growth conditions can alter P11 expression (Kaletsky et al., 2025), but whether low levels of P11 could permit F1 inheritance but not F2, is unknown.

Behavioral assays are notoriously finicky, not because they measure effects that are not robust, but because *C. elegans* are highly attuned to their environments; they integrate a myriad of environmental signals into an appropriate response. Hunter and colleagues tested potential sources of variability in the laboratory environment, including the sources of bacterial and worm strains, but none of these explained the discrepancy in their findings. The sources of variability identified by the Murphy lab (Kaletsky et al., 2025) provide a logical explanation for the differences between the two studies, but other unidentified environmental or procedural differences may also have contributed. The results of the Vidal-Gadea group, therefore, represent an important validation of the work of the Murphy group, and support the idea that procedural modifications made by the Hunter group contributed to their inability to observe transgenerational epigenetic inheritance.
